# The emergence of adolescent onset pain hypersensitivity following neonatal nerve injury

**DOI:** 10.1186/1744-8069-8-30

**Published:** 2012-04-24

**Authors:** David Vega-Avelaira, Rebecca McKelvey, Gareth Hathway, Maria Fitzgerald

**Affiliations:** 1 UCL Department of Neuroscience, Physiology & Pharmacology, University College London, Gower Street, London, WC1E6BT, UK; 2 Current address: School of Biomedical Sciences, University of Nottingham Medical School, Queens Medical Centre, Nottingham, NG7 2UH, UK; 3 Current address: Dpto. Fisiología, Edificio Medicina, Universidad Alcalá, Madrid, 28871, Spain

**Keywords:** Neuropathic pain, Microglia, Astrocytes, Spinal cord, Dorsal root ganglia, Neonatal

## Abstract

**Background:**

Peripheral nerve injuries can trigger neuropathic pain in adults but cause little or no pain when they are sustained in infancy or early childhood. This is confirmed in rodent models where neonatal nerve injury causes no pain behaviour. However, delayed pain can arise in man some considerable time after nerve damage and to examine this following early life nerve injury we have carried out a longer term follow up of rat pain behaviour into adolescence and adulthood.

**Results:**

Spared nerve injury (SNI) or sham surgery was performed on 10 day old (P10) rat pups and mechanical nociceptive reflex thresholds were analysed 3, 7, 14, 21, 28, 38 and 44 days post surgery. While mechanical thresholds on the ipsilateral side are not significantly different from controls for the first 2–3 weeks post P10 surgery, after that time period, beginning at 21 days post surgery (P31), the SNI group developed following early life nerve injury significant hypersensitivity compared to the other groups. Ipsilateral mechanical nociceptive threshold was 2-fold below that of the contralateral and sham thresholds at 21 days post surgery (SNI-ipsilateral 28 (±5) g control groups 69 (±9) g, p < 0.001, 3-way ANOVA, n = 6 per group). Importantly, no effect was observed on thermal thresholds. This hypersensivity was accompanied by macrophage, microglial and astrocyte activation in the DRG and dorsal horn, but no significant change in dorsal horn p38 or JNK expression. Preemptive minocycline (daily 40 mg/kg, s.c) did not prevent the effect. Ketamine (20 mg/kg, s.c), on the other hand, produced a dose-dependent reversal of mechanical nociceptive thresholds ipsilateral to the nerve injury such that thresholds return to control levels at the highest doses of 20 mg/Kg.

**Conclusions:**

We report a novel consequence of early life nerve injury whereby mechanical hypersensitivity only emerges later in life. This delayed adolescent onset in mechanical pain thresholds is accompanied by neuroimmune activation and NMDA dependent central sensitization of spinal nociceptive circuits. This delayed onset in mechanical pain sensitivity may provide clues to understand the long term effects of early injury such as late onset phantom pain and the emergence of complex adolescent chronic pain syndromes.

## Introduction

It is well documented that neuropathic pain is absent or transient in the youngest infants or children. Brachial plexus injuries at birth, followed by nerve repair, are not associated with pain [1] and reports of phantom pain, complex regional pain syndrome, peripheral neuropathy pain are very rare before 5–6 years of age [2]. This is supported by animal models of neuropathic pain, spared nerve injury (SNI) and chronic constriction injury (CCI), where little or no allodynia and pain behaviour is observed in animals whose nerves have been injured before three weeks of age or postnatal day 21 (P21) [3-5].

The aim of the current study was to investigate the longer term effects of early nerve injury and to test whether pain or hypersensitivity might emerge beyond the post injury period, perhaps only in adolescence. To date, the longer term effects of nerve injury, and the possibility of emergence of pain beyond childhood has not specifically been addressed [6-8]. Most studies of nerve injury in infants have not followed up the long term consequences; obstetric injuries in man have been followed up for only for about 7 years and experimental nerve injury rat pups, only for a few weeks [5]. Intriguing clues exist in the clinical literature that symptoms may appear later in life. Thus, when Melzack and colleagues studied phantom limb pain in adolescents with either congenital or early surgical loss of limbs, they noted that onset of phantoms in those with the earliest loss were remarkably delayed – emerging only after a mean of 7 years [9]. The possibility that nerve injury in childhood could cause a delayed onset pain syndromes is interesting in view of the onset of unexplained pain syndromes such as complex regional pain syndrome (CRPS) in adolescence [10].

We have carried out a longitudinal study of pain behaviour and immune responses into adulthood, following “spared nerve injury” (SNI) in young rats. We have observed that while young rats fail to display neuropathic behaviour for the first weeks after nerve injury, at adolescence they develop lasting cutaneous mechanical hypersensitivity as a consequence of this early-life nerve injury.

## Materials and methods

### Surgery

Surgery was performed on adult (8 weeks old) male Sprague–Dawley rats and rat male pups (10 days postnatal age, P10) and carried out in accordance with the United Kingdom Animal (Scientific Procedures) Act 1986. Spared nerve injury (SNI) and sham surgery were performed under (4 %) halothane general anaesthesia with antiseptic conditions as described previously [11]. After skin preparation, the sciatic nerve and its three terminal branches were exposed in the upper lateral thigh. The common peroneal and tibial branches were cut and ligated under direct vision, leaving the sural nerve intact. Muscle and skin were closed in two layers. In sham operations, the procedures were the same but the nerves were only exposed and not cut or ligated. In all cases great care was taken neither to stretch the nerve or its branches nor to damage the intact nerves.

After surgery, animals were returned to their cages and litters and maintained on a 12 hour light/dark cycle at constant ambient temperature with free access to food and water until the next procedure (behaviour or tissue collection for immunostaining).

### Behavioural testing

#### *Mechanical pain thresholds*

Rats that had undergone spared nerve injury (SNI) or sham surgery at P10 were tested for cutaneous mechanical sensitivity at 3, 7, 14, 21, 28, 38 and 44 days post surgery (n = 6 per group). Flexion withdrawal reflex thresholds in response to punctate mechanical stimulation of the dorsolateral surface of the hindpaw was tested using calibrated von Frey filaments (VF), that exert a reproducible stimulus strength in grams (Stoelting, Woodvale, IL). The dorsolateral surface of the hindpaw is innervated by the sural nerve, and this test is known to reveal mechanical allodynia in the adult rat after SNI surgery [11]. Filaments were applied sequentially to the plantar surface of the hind paw six times at intervals of 1 s. Response threshold was defined as the VF filament which produced reflex paw withdrawal in 4 out of 6 applications.

#### *Thermal pain thresholds*

Rats that had undergone spared nerve injury (SNI) or sham surgery P10, were also tested for thermal cutaneous sensitivity at 3, 7, 14, 21, 35, 45 and 51 days post surgery (n = 6 per group). The lateral plantar surface was exposed to a beam of radiant heat through a transparent Perspex surface (Hugo Basile Inc.) [12]. The flexion withdrawal reflex latency was recorded, with a minimal value of 0.5 s and a maximum of 15 s. The heat stimulation was repeated 3 times at an interval of 5–10 min for each paw and the mean calculated.

#### *Minocycline administration*

Rat pups (n = 6) received a 40 mg/kg i.p. minocycline, a dose which reverses mechanical allodynia after nerve injury in adults [13]. The first injection was administered the day before (P9) SNI surgery, the second just prior to surgery at P10 and this was followed by daily ip injections of minocycline until 21 days post surgery (P31). Two control groups were used (i), a group of SNI animals injected with saline (n = 6), and (ii) a group of sham surgery animals, injected with either saline (n = 6) or minocycline (n = 6). The animals were tested for mechanical pain thresholds using calibrated von Frey hairs. The test was carried out by a blinded observer: the animals were tagged with a chip applied subcutaneously to avoid any identifiable external marks and the animals were given to the observer in a random order. The data was collected for analysis by ANOVA.

#### *Ketamine administration*

Young adult rats (P31) that had undergone SNI (n = 6) or sham (n = 6) surgery at P10 were treated with subcutaneous ketamine (Sigma) in cumulative doses of 1, 10 and 20 mg/Kg in a volume of 250 μl as described elsewhere [14]. Doses were administered at intervals of one hour. Two methods were used: 1) Change in response frequency to a threshold mechanical stimulus, where the frequency of paw withdrawal before and after ketamine injection in response to the same, threshold von Frey hair applied to the dorsolateral area of the paw, was compared. The mean mechanical threshold was significantly different in the ipsilateral SNI group (31 ± 6 g) and the control groups (67 ± 4 g), (ANOVA p < 0.01, Tukey p < 0.05). The data is expressed as a percentage of responses to the corresponding baseline mechanical threshold, plotted using average and standard error of the mean and statistically analysed using a 2-way ANOVA. 2) Change in mechanical pain thresholds measured with a dynamic plantar aesthesiometer (Ugo Basile). Animals were habituated in Perspex cages with a mesh floor and the aesthesiometer probe applied in the lateral plantar surface of the paw within the territory innervated by the sural nerve [11]. The intensity of the stimulus increased from 0 to 50 g in 30 seconds and the average threshold taken from two applications. Mechanical sensitivity thresholds were established before (baseline) and one hour after each dose administration and a final measurement was performed at 72 hours post ketamine administration (after ketamine clearance).

### Immunostaining

Tissue was extracted from rats that had undergone surgery at P10 or as adults at 7 and 30 days (P10 only) post surgery. Rats were overdosed with 100 mg/kg of Euthanal® and perfused through the heart with 200 ml heparin-saline, followed by 200 mL 4 % paraformaldehyde in 0.1 M phosphate buffer (PB). The L4–L5 lumbar spinal cord and the corresponding L4 and L5 ganglia were removed and postfixed in 4 % paraformaldehyde for 1 h at 4°C followed by immersion in 30 % sucrose in 0.1 M of PB at 4°C for at least two days before sectioning. Serial, 20 μm cryostat sections were cut and stained after blocking in 10 % normal goat serum, 0.3 % Triton X in 1× PBS using primary antibodies: Rabbit anti-IBA-1 (1/1000, Wako®), mouse anti NF200 (1/1000, Chemicon®), and rabbit-anti-GFAP (1/100 Dako®). The antibodies were incubated overnight at room temperature in 3 % normal goat serum, 0.3 % Triton X in 1× PBS. Secondary antibodies were goat anti-mouse (Alexa-488®) and goat anti-rabbit (Alexa-593®) at 1/200 dilution in 3 % normal goat serum, 0.3 % Triton X in 1× PBS. Antibody excess was removed by 3 washes in PBS for 10′ at RT. The isolectin IB4 (*Bandeira simplicifolia)* labelled with FITC (Sigma®) was used to identify the non-peptidergic neurons [15]. After staining, the sections were kept for 16 h in the dark to normalize the background and then microscope images acquired at 10X magnification with OpenLab® software at a constant exposure time of 1.02″.

The images generated by immunohistochemistry were analysed with *Image J 1.36* (NHS) software, n = 3 rats per experimental group, n = 4-6 sections spinal cord or ganglia per animal. In the spinal cord, microglia (IBA-1 positive; [16] or astrocytes (GFAP positive; [17]) were counted in the region of loss of IB4 staining, which delineates the area of termination of lesioned nerve in the SNI model on the ipsilateral and equivalent contralateral side [18]. Data is expressed as fold difference of the contralateral side and analysed using 2-way ANOVA and plotted using average and standard error of the mean.

In the ganglion, sections were stained with IBA-1 to label macrophages [19] and with NF200 to label large neurons [20]. Representative sections from the centre of the ganglion, 160 μm apart, were used for analysis. Macrophage activation was assessed as previously described [21], by counting neurons with enlarged macrophages and processes clustering around the NF200 + ve neuronal cell bodies in characteristic 'ring-like' structures. Data was expressed as percentage of large NF200 positive neurons with IBA-1 positive macrophage ring-like structures and analysed using 2-way ANOVA and plotted using average and standard error of the mean.

### Western-blot

The dorsal horn quadrant (ipsilateral or contralateral) of L3–L5 spinal cord segments was snap frozen in liquid nitrogen after extraction and stored at -80^o^ C until further procedure. Proteins were extracted in 150 μL RIPA buffer (NP-40 1 %, Hepes 20 mM, pH 7.2, NaF 100 mM; NaCl 100 mM, NaVO 1 mM, EDTA 5 mM, and 1 % of protease inhibitor) from Sigma by blender homogenisation, incubated for 2 h on ice and centrifuged at 12,000 g to remove the debris. The proteins were diluted to yield 1 mg/mL and stored in aliquots at -80^o^ C. 10 μg of total protein were used for the electrophoresis with the Mini Protean 3 Cell System (Bio-Rad) with 10 % acrylamide precast Ready gels (Bio-Rad). Electrophoresis was carried out in Tris 20 mM, glycine 247 mM, and 0.0001 % SDS buffer at 4^o^ C until the reference colorant reached the end of the gel. The Rainbow coloured protein ladder from Amersham was loaded to monitor the electrophoresis. The transference blots to the PVDF membranes (Bio-Rad) were done in Tris 48 mM, glycine 77 mM, 0.0001 % SDS, and 10 % of methanol for 1.5 h at 4^o^ C. Following the blot, membranes were blocked in PBS solution (PBS tablets, Sigma) with 0.1 % Tween 20 (Sigma) and 4 % semi-skimmed milk powder (Sainsbury’s). The primary antibodies were incubated at 4^o^ C overnight with gentle shaking and diluted in blocking buffer (rabbit anti-phospho-p38 or rabbit anti-phospho-JNK at 1:1000 from Cell Signaling and mouse anti-GAPDH from Chemicon at 1/2000). Secondary antibodies from Santa Cruz were used at a dilution of 1/2000. Antibody excess was removed with six washes in PBS/0.1 % Tween 20. To reveal the signal we used the ECL western blotting detection system from Amersham and the images were acquired with the Quantity One system (Bio-Rad). The intensity of the western blots bands for phospho-p38 (~40 kDa), phospho-JNK (~46 and ~54 Kda bans) and GFAP (~50 kDa) were measured with Quantity One, v4.6.2 (Bio Rad) densitometry using GAPDH (36 kDa band) housekeeping gene for normalization. Values were expressed as the ratio “densitometry protein/densitometry GAPDH”.

## Results

### Delayed adolescent onset of mechanical hypersensitivity following early-life nerve injury

To analyse the long term consequences of early-life nerve injury in pain behaviour we carried out a longitudinal study of rats that had undergone spared nerve injury (SNI) or sham operations when they were 10 days old (P10).

**Figure 1:**
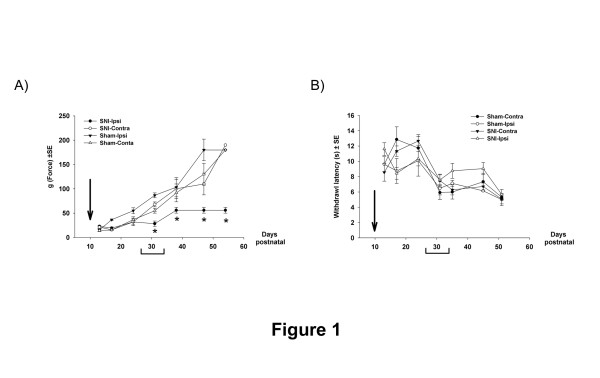
Nerve injury at the age of 10 days has no effect over the first 2–3 weeks but gradually ipsilateral mechanical hypersensitivity emerges in adolescence (3-way ANOVA, p < 0.001; post hoc Tukey, p < p.05, n = 6 per experimental group). **B**) Thermal pain thresholds show a general developmental reduction in latency in all experimental groups with no significant differences in the SNI group when compared to sham group.

Mechanical nociceptive reflex thresholds were analysed 3, 7, 14, 21, 28, 38 and 44 days post P10 surgery using calibrated von Frey filaments (VF). As previously reported [5], mechanical thresholds increase steadily with increasing postnatal age in the control sham group (ipsilateral and contralateral side) and on the unoperated contralateral side of the SNI animals (Figure 1A), where threshold rises from 18 (±2) g at P13 (3 days post surgery) to 180 g at P54 (44 days post surgery). Figure 1A shows mechanical thresholds on the ipsilateral side to SNI surgery are not significantly different from controls for the first 2–3 weeks post P10 surgery, in agreement with previous studies [5]. However, Figure 1A also shows that after that time period, beginning at 21 days post surgery (P31), the SNI group developed a significantly mechanical hypersensitivity compared to the other groups. At 21 days post surgery, the ipsilateral mechanical nociceptive threshold falls by 2-fold below that of the contralateral and sham thresholds (SNI-ipsilateral 28 (±5) g control groups 69 (±9)g, p < 0.001, 3-way ANOVA, n = 6 per group). By 28 days postsurgery (P38), the SNI-ipsilateral threshold reaches a plateau of 56 (±6)g, which is 1.7 fold lower than control groups and this remains significantly lower by 1.9 fold and 3.3 fold at P48 and P54 respectively; (p < 0.001, 3-way ANOVA, Figure 1A).

Figure 1B shows the same longitudinal study on radiant heat nociceptive thresholds, post P10 SNI surgery. As reported elsewhere [22,23], thermal thresholds tend to fall with postnatal age. However, in contrast to the effect on mechanical nociception, analysis of thermal nociceptive thresholds shows no significant differences in the SNI group when compared to the control groups throughout the longitudinal study up to P54. (Figure 1B).

### Early-life nerve injury evokes a delayed adolescent onset glial activation

In the light of the known relationship between glial activation and hyperalgesia [24-26], we analysed the time course of activation of microglia and astrocytes in the spinal cord and macrophages in dorsal root ganglia following early life nerve injury [27-29]. Glial activation was examined in rats, which had undergone SNI surgery at P10 rat pups, at two postnatal time points. The first was at 7 days post surgery (P17), when there was no change in mechanical sensitivity and the second was at 21 days post surgery (P31) when mechanical hypersensitivity had developed and both were compared with sham groups. In addition, we included a group of rats that underwent surgery as adults, 7 days post surgery as a positive control of glia activation [30].

#### *Microglia*

**Figure 2:**
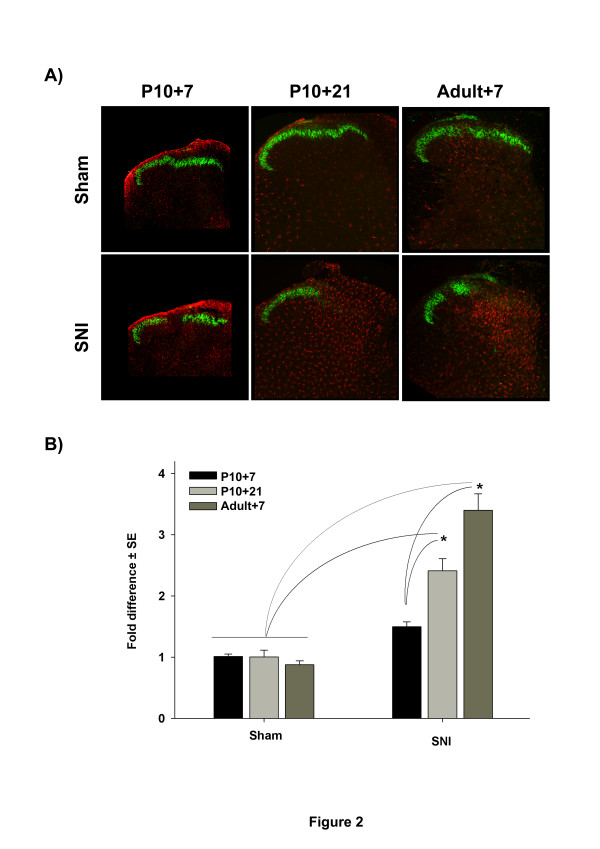
Shows sections through the L4 dorsal horn at various times after sham (top) and SNI (bottom) surgery at P10 (left and middle) and adult (right). Sections are immunostained with the microglia marker IBA-1 (red) and the non peptidergic C-fibre marker isolectin IB-4 (green). SNI surgery at P10 produces non significant microglia activation after 7 days compared to sham in (P10) rats. However, there is a late onset of microglia activation at 21 days post surgery which is significantly greater than sham and similar to that seen in adult rats 7 days post surgery. The gap in isolectin IB-4 is a marker of the terminal region of the nerves damaged by SNI surgery. **B**) Quantification of microglia activation in the three experimental groups illustrated in A. IBA-1 stained microglial cells were counted and the results are expressed as fold difference of the contralateral side. *: (n = 3 animals per experimental group, 3-way ANOVA, p < 0.001, Tukey, p < 0.05).

Figure 2 shows the effects of SNI surgery upon spinal microglia activation. SNI surgery at P10 has no effect on microglial activation in the lumbar dorsal horn 7 days post surgery (P17), as reported previously [30,31], but at 21 days post surgery (P31) there is significant activation of microglia in the dorsal horn, (p < 0.001, 2-way ANOVA) (Figure 2A and 2B). Figure 2A shows the distribution of microglia (stained with the specific marker IBA-1, in red) in the L4/L5 lumbar region of the spinal cord. The depletion of isolectin IB4 (in green) from non-peptidergic C-fibre terminals [32,33] delineates the termination area of lesioned nerves. The immunostaining shows a clear increase in microglia cell numbers (proliferation) ipsilateral to the nerve injury group compared to control groups at 21 but not at 7 days post P10 surgery (Figure 2A). Quantification of microglia staining shows that 7 days post P10 surgery there is no significant microglia activation when compared to the control groups, while 21 days post P10 surgery there is a significant increase (2.4 ±0.2 fold, p < 0.001, 2-way ANOVA) in microglia activation comparable to that seen in adult rats, post surgery (3.4 ±0.3) (Figure 2B). In the adult injured group, microglia distribution is largely restricted to the termination area of damaged afferents, as denoted by the IB4 staining as reported elsewhere [34]. In contrast, delayed microglial activation following P10 nerve injury was spread more widely throughout the whole ipsilateral dorsal horn, at 21 days post surgery (Figure 2A).

#### *Astrocytes*

**Figure 3:**
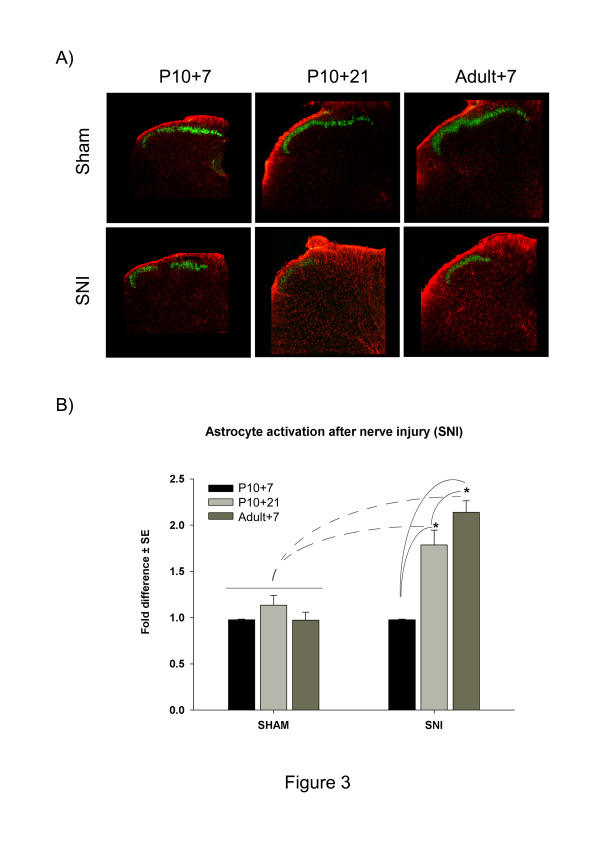
Shows sections through the L4 dorsal horn at various times after sham (top) and SNI (bottom) surgery at P10 (left and middle) and adult (right). Sections are immunostained with the astrocytic marker GFAP (red) and the non peptidergic C-fibre marker isolectin IB-4 (green). SNI surgery at P10 produces non significant astrocyte activation after 7 days compared to sham in (P10) rats. However, there is a late onset of astrocyte activation at 21 days post surgery which is significantly greater than sham and similar to that seen in adult rats 7 days post surgery. The gap in isolectin IB-4 is a marker of the terminal region of the nerves damaged by SNI surgery. **B**) Quantification of astrocyte activation in the three experimental groups illustrated in A. GFAP stained astrocytes with signs of gliosis were counted and the results are expressed as fold difference of the contralateral side. *: (n = 3 animals per experimental group, 3-way ANOVA, p < 0.001, Tukey, p < 0.05).

Figure 3 shows the effects of surgery upon spinal astrocyte activation, using GFAP immunostaining to quantify astrogliosis (increase in cell numbers with larger cell bodies and processes) [17]. Figure 3A, shows that rats that undergo SNI surgery at P10, show a clear increase in GFAP immunoreactivity 21 days post surgery which is not observed 7 days post surgery. Quantification of GFAP immunoreactivity shows a significant (p < 0.001, 2-way ANOVA) increase (1.7 ±0.2 fold) in astrogliosis 21 days post early-life nerve injury (at P10) which is not seen at 7 days. This pattern of astrocyte activation is comparable to the astrogliosis observed after nerve injury in adult rats (2.1 ± 0.1 fold) (Figure 3B).

#### *Macrophages*

**Figure 4:**
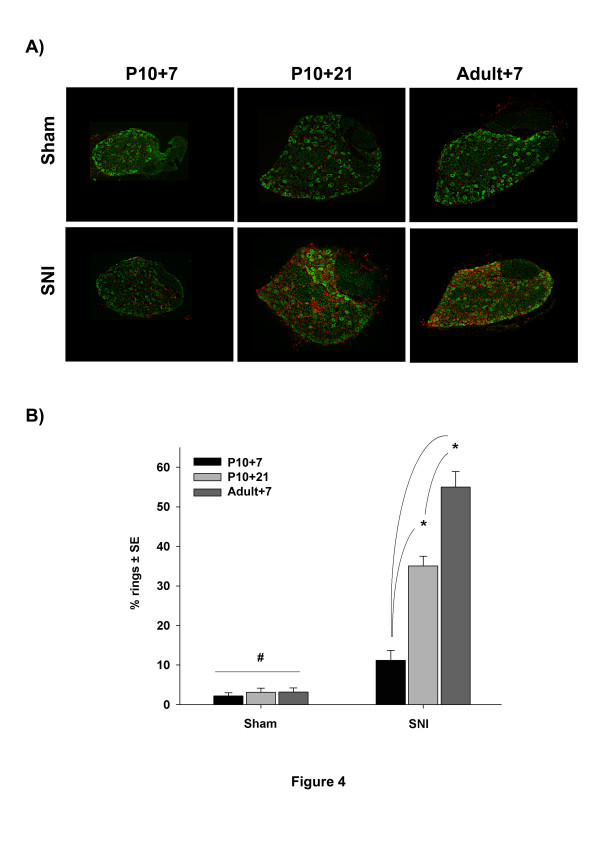
Shows sections through the L4 dorsal root ganglion at various times after sham (top) and SNI (bottom) surgery at P10 (left and middle) and adult (right). Sections are immunostained with the macrophage marker IBA-1 (red) and the large A cell neuronal population marker NF200 (green). Macrophage activation is observed as macrophages clustering around large neuronal cell bodies in characteristic ‘ring-like’ structures with processes extending from their enlarged cell bodies towards large neurons. SNI surgery at P10 produces non significant macrophage activation after 7 days compared to sham in (P10) rats. However, there is a late onset of macrophage activation at 21 days post surgery which is significantly greater than sham and similar to that seen in adult rats 7 days post surgery. **B**) Quantification of these activated macrophage ‘ring-like’ structures in the three experimental groups illustrated in A and expressed as a percentage accordingly to large sensory neurons*: (n = 3 animals per experimental group, 3-way ANOVA, p < 0.001, Tukey, p < 0.05).

Figure 4 shows the effects of surgery upon IBA-1 positive macrophage activation in the dorsal root ganglion. Again, at 7 days post P10 nerve injury there was no significant change in macrophage distribution or morphology when compared to control groups. In contrast, at 21 days post P10 surgery, clear macrophage activation is observed, where macrophages (in red) cluster around large neuronal cell bodies in characteristic ‘ring-like’ structures with processes extending from their enlarged cell bodies towards large neurons (in green) (Figure 4A). Quantification of these macrophage ring-like structures show a significant (p < 0.001, 2-way ANOVA) increase at 21 days post surgery (35 % ± 3) but not at P17 (11 % ±3). This increase in macrophage activation is below the 55 % ± 4 activation observed in adult injured rats but is still significantly (p < 0.001, 2-way ANOVA) greater than control groups (2-3 %), (Figure 4B).

### Minocycline fails to prevent adolescent mechanical hypersensitivity produced by early-life nerve injury

**Figure 5:**
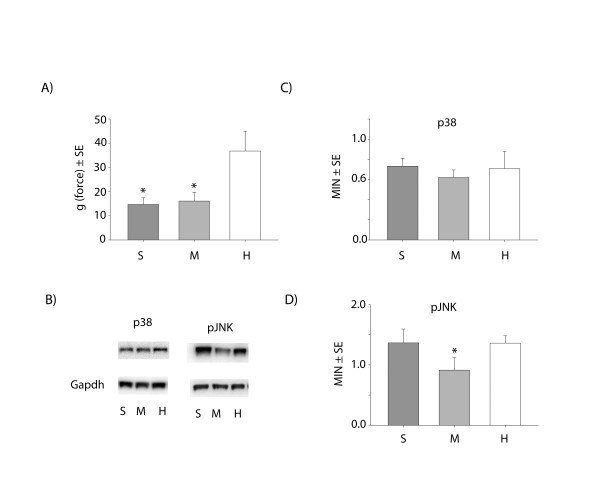
Mechanical pain thresholds in animals with early-life (P10) nerve injury (S) or sham surgery H) tested in later life (P31). Mechanical pain thresholds were measured in minocycline (M) (40 mg/Kg daily ip injections) after SNI nerve injury compared to SNI saline (S) group and controls (H). n = 6 per experimental group; *: ANOVA, p < 0.001, Tukey, p < 0.05 **B**) Representative images of western-blot for phospho-p38 and phospho-JNK (pJNK) and the housekeeping gene Gapdh. Quantification was done by densitometry of p38 (**C**) and pJNK(**D**). *: n = 4 per experimental group; *: ANOVA, p < 0.001, Tukey, p < 0.05.

To test whether late onset mechanical hypersensitivity produced by P10 nerve injury is caused by delayed adolescent onset of glia activation, we administered the microglial inhibitor minocycline to sham and SNI rat pups [13]. P10 rats underwent daily i.p. minocycline (40 mg/kg) injections, beginning the day before SNI surgery (P9), again immediately prior to SNI surgery at P10, and then daily until 21 days post surgery (P31). Figure 5A shows that at P31 there was no difference in the mechanical sensitivity of the SNI-minocycline (M) treated animals (16.1 ±4 g force) when compared to a SNI-saline (S) treated group (14.6 ± 2.7 g force, Figure 5A). The mechanical threshold was significantly lower in both minocycline and saline SNI groups (ANOVA, p < 0.01; Tukey, p < 0.05) than the sham group (H) (37.5 ± 2.1 g force).

In adult models of nerve injury induced neuropathic pain, microglial activation is accompanied by phosphorylation of p38 and this is well known marker of microglia activation after adult nerve injury [28]. Furthermore, this phosphorylation of p38 after adult nerve injury is known to be inhibited by minocycline [35,36]. However, interestingly, we did not find any differences in dorsal horn phospho-p38 levels between the SNI-saline and sham groups 21 days post surgery (P31), despite the presence of activated microglia and behavioural hypersensitivity (Figure 5). Figure 5 also shows the phospho-p38 levels in SNI-minocycline treated animals are not significantly different to the SNI-saline or sham groups (Figure 5B and C).

We also analysed the phosphorylation of c-Jun N-terminal kinase (JNK) which is a marker of astrocyte activity after nerve injury and required for maintenance of neuropathic pain [37,38]. We did not find any significant difference when comparing the SNI-saline and sham groups (Figure 5C and D) but minocycline had a small (1.5 fold) but significant effect up on the SNI group p < 0.001, 1-way RM-ANOVA, n = 4 per group).

### The NMDA antagonist ketamine reverses adolescent onset mechanical hyperalgesia produced by early-life nerve injury

**Figure 6:**
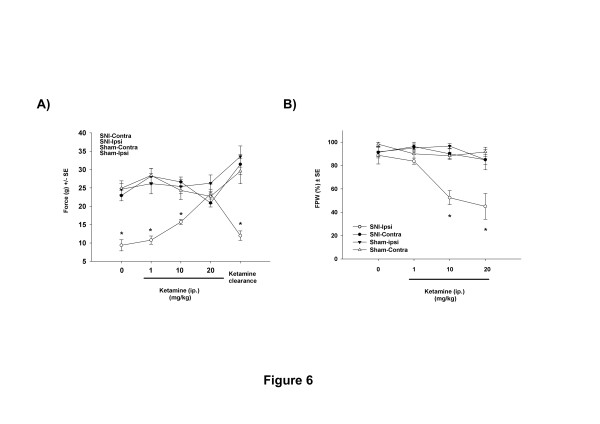
Mechanical pain thresholds in animals with early-life (P10) nerve injury (SNI-ipsi) or sham surgery (sham-ipsi) tested in later life (P31), before and after i.p ketamine. SNI surgery animals have lower baseline mechanical pain thresholds but the NMDA antagonist ketamine increases mechanical pain thresholds to normal levels in a dose dependant manner. The clearance of ketamine (72 h post injection) results in the mechanical pain thresholds falling back to pre-ketamine levels. **B**) The same dose dependent reversal of mechanical hypersensitivity by ketamine is observed when measuring foot paw withdrawal to a von Frey hair. *: (n = 6 per experimental group, 3-way ANOVA, p < 0.001, Tukey, p < 0.05), SNI: spared nerve injury, Ipsi: ipsilateral, Contra: contralateral, ip: intraperitoneal, FPW: frequency of paw withdrawal. Ketamine had no effect upon contralateral (SNI-contra) thresholds or upon sham operated animals.

To analyse whether the late adolescent onset mechanical hypersensitivity is due to classic NMDA-dependent central sensitization mechanisms [39], we tested the effect of ketamine, the NMDA antagonist, upon the mechanical hyperalgesia evoked by early life nerve injury. Rats that underwent SNI surgery at P10 were tested 21 days post surgery (P31) when they had developed clear and significant differences in mechanical nociceptive thresholds. Figure 6A shows the effect of treatment with cumulative doses of ketamine (0, 1, 10, 20 mg/kg, s.c) upon the mechanical hypersensitivity. The results show a clear dose-dependent reversal of mechanical nociceptive thresholds ipsilateral to the nerve injury (9 ±1, 10 ±1, 15 ±1 and 23 ±1 grams-force at 0, 1, 10 and 20 mg/Kg of ketamine respectively) such that they reach control levels (26 ±1 grams-force) at the highest doses of 20 mg/Kg (Figure 6A). The clearance of the ketamine (72 h after injection) eliminates its effect and the thresholds fall again to 12 ±1 grams-force (Figure 6A).

The ability of ketamine to reduce mechanical hypersensitivity was also measured using the frequency of paw withdrawal threshold after mechanical noxious stimulation with von Frey filaments (VF). The results show a significant (p < 0.001, 3-way ANOVA, n = 6 per group) reduction in the frequency of paw withdrawal at the ketamine doses of 10 mg/Kg (to 52 % ±6) and 20 mg/Kg (to 45 % ±11) (Figure 6B).

Ketamine had no effect on mechanical sensitivity contralateral to the nerve injury or in the sham controls in both tests (Figure 6A and 6B).

## Discussion

In this study we have analysed the long term consequences of early-life nerve injury upon pain behaviour in the rat. We have demonstrated for the first time that early life nerve damage produces a mechanical hypersensitivity that only emerges in adolescence. In contrast to the rapid and profound allodynic effects in adults [11,40], spared nerve injury at postnatal day 10 has little or no effect upon pain behaviour in the first weeks after surgery. However, if threshold testing is extended to 3 weeks after the surgery, a mechanical hypersensitivity on the ipsilateral hindpaw emerges which is maintained into adult life. This hypersensitivity was not observed on the contralateral paw or following sham injury. Moreover, no differences were observed in thermal nociceptive thresholds in these animals ruling out a non-specific motor deficit and suggesting that early life spared nerve injury has a specific effect upon adult mechanical nociception.

Mechanical pain thresholds steadily increase through postnatal development due to fine tuning of spinal pain circuits [41] and the maturation of spinal inhibitory networks [42]. This steady rise is also observed in animals after nerve lesion [5] but only while the animal is still young. However, at the onset of the adolescence, from P30 onwards [43], this steady rise stops on the nerve injured side such that a significant 2-fold decrease in mechanical pain thresholds emerges leaving the adult animal with a selective mechanical, but not thermal hypersensitivity on the affected side.

Clinically, there are few studies on the long term effects of peripheral nerve injury in infants. While neuropathic pain is not reported in young patients following infant brachial nerve plexus avulsion [1,44], self-mutilation is more frequent in these children [45] due to either the subsequent post trauma surgery or the initial nerve injury or both. Perhaps more relevant, it has been reported that the appearance of phantom syndromes (many of which are painful) following loss of limbs in infancy, do not appear for many years after the original loss, in some cases up 3 to 15 years later [9,46]. This delayed onset pain after early nerve injury may also contribute to chronic complex pain syndromes with cutaneous sensory abnormalities which arise in adolescence, but whose aetiology is unknown [10,47]. The animal model described here might cast some light on such syndromes, revealing as it does, the possibility for hypersensitivity to arise a considerable time after an original injury in infancy.

One possible mechanism underlying this emergence of mechanical sensitivity might lie in the neuroimmune system. Glial activation is a common characteristic of adult neuropathies [25,26] and several studies have demonstrated the need for microglial/macrophage activation to initiate the neuropathy in the adult rodent [13,27,28]. We have previously demonstrated that there is little activation of either both spinal microglia [30,31] or macrophages in the dorsal horn [21] in the young P10 rat after 7 days post nerve injury and our recent transcriptome analysis of the dorsal root ganglia and the dorsal horn at different ages reveals a much greater and different profile of immune related genes regulated in the adult compared to young rats after nerve injury [21,48]. Since it is well known that the immune system goes through dramatic changes of maturation through the postnatal development [49,50], we hypothesized that a late onset change in microglia/macrophage activation was the trigger for the emergence of mechanical hypersensitivity in adolescence. Consistent with this proposal, our results showed that microglia, astrocytes and macrophages are only activated after early life nerve injury at the onset of the adulthood (at P30), the same time point at which mechanical hypersensitivity emerges. However, importantly, microglial activation was not accompanied by an increase in phosphorylated p38 expression at the onset of the adulthood (at P30) after early life nerve injury when compared to the baseline levels of phosphorylation in the sham operated group. While p38 phosphorylation is a key event in microglia activation when the nerve injury is sustained in adult animals[28], our results indicate that the p38 map kinase pathway is not involved in this late onset of microglia activation after early in life nerve injury. This would explain why preemptive minocycline (a p38 inhibitor [35,36]), at a dose designed to maximise its suppression of macrophage/microglial activation [13] did not prevent or even reduce the delayed onset adolescent mechanical hypersensitivity.

It has been proposed that astrocytes have a key role in the maintenance of neuropathic pain in adults [38] and that microglia activation is required for the activation of astrocytes [51,52]. Interestingly, we also observed astrogliosis at onset of adulthood (P30), but similarly to microglia, we did not find differences in JNK phosphorylation (a marker of astrocyte activation) at the onset of the adulthood (at P30) after early life nerve injury when compared to the baseline levels of phosphorylation in the sham operated group. The small effect of minocycline upon JNK phosphorylation was evidently not sufficient to ameliorate the mechanical hyperalgesia.

Despite the rise in activation of microglia and astrocytes at the onset of the adulthood (at P30) after early life nerve injury, our results suggest that this activation must involve different signalling pathways to those classical map kinase pathways found when the injury is sustained in the adulthood [28,37]. We cannot rule out the possibility of a different role for glia in this model of early in life nerve injury. In this way, microglia is a heterogeneous group with many functions that include surveillance (resting microglia), toxicity (classical activation associated with pro-inflammatory cytokines), neuroprotection (alternative activation associated with anti-inflammatory cytokines and repair) [53-55]. Thus, it could be possible that glia has a different role in this model where microglia activates towards a healing process with the astrocyte support. Our results show that microglia is not activated after nerve injury as it would be expected for a repair phenotype. Instead, microglia activation begins at the onset of adult hood 3 weeks after the initial insult and is accompanied by mechanical hypersensitivity. The experimental models of neuropathic pain in adult clearly show that microglia activation has pro-inflammatory phenotype [56,57] and this activation is directly linked to initiation of pain behaviours [13]. Thus, our results are more in accordance with a role of microglia in triggering mechanical hypersensitivity. Nevertheless, we consider that further analysis is required to understand the role and mechanisms of microglia and astrocytes activation in this model of early in life nerve injury.

To explore the neuronal mechanisms that might underlie this novel mechanical hypersensitivity following early life nerve injury we have explored a classic mechanism that underlies maintained neuropathic mechanical allodynia in adult nerve injury models, namely NMDA mediated central sensitization [58]. Central sensitization is a well-established mechanism whereby dorsal horn neuronal activity is enhanced due to increased membrane excitability as a result of opening voltage sensitive NMDA channels allowing Ca^2+^ entry into the cell [59]. Activation of NMDA receptors is an essential step in both initiating and maintaining activity-dependent central sensitization [60] and the use of inhibitors such us ketamine can attenuate neuropathic allodynia in adults [14,61]. In the present study, we have clearly demonstrated that ketamine applied when the rat reaches adulthood reverses the mechanical hypersensitivity following early life nerve injury to baseline levels. The mechanism by which this central sensitization only emerges three weeks after P10 nerve injury is not clear, but it is possible that it results from alterations in activity dependent maturation of dorsal horn inhibitory circuits [62] or descending controls of dorsal horn excitability [63].

The importance of early life events in individual disease susceptibility is well known but recognition that early pain experience may also predispose individuals to greater pain at a later stage of life is a more recent concept. Increasing evidence of the activity dependence and plasticity of both peripheral and central sensory connections in the neonatal period suggests that excess nociceptive input or tissue injury in infancy could lead to prolonged structural and functional alterations in pain pathways lasting into adult life [23,64]. This is especially relevant to individuals who have experienced significant and repeated noxious stimulation as a result of surgery and intensive care in infancy [11]. Such clinical procedures evoke robust behavioural and physiological responses at spinal cord and cortical level [4,53] causing measurable hyperalgesia [5,6,52] and may leave a lasting susceptibility to pain in later life [2]. The effects we report here are restricted to early life infant nerve injury which causes no sign of pain in the weeks, months and years after injury in humans and the same equivalent developmental period in rats. However, adolescence reveals a baseline hypersensitivity after early in life nerve injury that is similar to that seen in humans. Therefore, we provide a new insight to those mechanisms that contribute to chronic pain behaviours such as late onset of phantom syndromes.

## Conclusion

In conclusion, we report here, a novel consequence of early life nerve injury whereby mechanical hypersensitivity only emerges later in life. This delayed adolescent onset in mechanical pain thresholds is accompanied by neuroimmune activation and NMDA dependent central sensitization spinal nociceptive circuits. This delayed onset in mechanical pain sensitivity may provide clues to understanding the long term effects of early injury such as late onset phantom pain and the emergence of complex adolescent chronic pain syndromes.

## Abbreviations

SNI, Spared Nerve Injury; (CCI), Chronic constriction injury; Ipsi, Ipsilateral side; Contra, Contralateral side; PHI, Young sham group, ipsilateral side; ip, Intraperitoneal; SE, Standard error; S, Spared nerve injury; M, Minocycline; H, Sham; MIN, Mean intensity normalised.

## Competing interests

The authors declare that they have no competing interests.

## Authors’ contributions

DVA designed the study, carried out all the experiments, performed the statistical analysis and wrote the manuscript. RM and GH carried out part of the behavioural analyses. MF is the director of the research and wrote the manuscript. All authors have read and approved the final manuscript.
